# Leukemia Stem Cells as a Potential Target to Achieve Therapy-Free Remission in Chronic Myeloid Leukemia

**DOI:** 10.3390/cancers13225822

**Published:** 2021-11-20

**Authors:** Kyoko Ito, Keisuke Ito

**Affiliations:** 1 Ruth L. and David S. Gottesman Institute for Stem Cell and Regenerative Medicine Research, Albert Einstein College of Medicine, Bronx, NY 10461, USA; kyoko.ito@einsteinmed.org; 2 Departments of Cell Biology and Stem Cell Institute, Albert Einstein College of Medicine, Bronx, NY 10461, USA; 3 Department of Medicine, Montefiore Medical Center, Albert Einstein College of Medicine, Bronx, NY 10461, USA

**Keywords:** chronic myeloid leukemia, leukemia stem cell, metabolic regulation, microenvironment

## Abstract

**Simple Summary:**

Leukemic stem cells represent a rare subpopulation of leukemic cells, which not only drive leukemia initiation and progression, but also contribute to drug resistance and/or disease relapse. To achieve permanent cures and prevent relapse, eradication of leukemia stem cells is essential. Chronic myeloid leukemia is a myeloproliferative disorder, and tyrosine kinase inhibitors have dramatically improved long-term outcomes and quality of life for patients. Point mutations of the kinase domain of *BCR-ABL1* lead to drug resistance, and as a result, several new generations of tyrosine kinase inhibitor have been introduced to the clinic. Some patients develop drug resistance without known mutations, however, and the presence of leukemia stem cells is believed to be at least partially associated with resistance development and disease relapse. The identification of specific markers distinguishing leukemia stem cells from healthy hematopoietic stem cells, and the potential contributions of the bone marrow microenvironment to leukemia pathogenesis, have also been explored. In this review, we revisit the current knowledge regarding the roles of leukemia stem cells in response to pharmacological treatment and explore how durable treatment-free remission may be achieved after discontinuing tyrosine kinase inhibitor treatment.

**Abstract:**

Leukemia stem cells (LSCs, also known as leukemia-initiating cells) not only drive leukemia initiation and progression, but also contribute to drug resistance and/or disease relapse. Therefore, eradication of every last LSC is critical for a patient’s long-term cure. Chronic myeloid leukemia (CML) is a myeloproliferative disorder that arises from multipotent hematopoietic stem and progenitor cells. Tyrosine kinase inhibitors (TKIs) have dramatically improved long-term outcomes and quality of life for patients with CML in the chronic phase. Point mutations of the kinase domain of *BCR-ABL1* lead to TKI resistance through a reduction in drug binding, and as a result, several new generations of TKIs have been introduced to the clinic. Some patients develop TKI resistance without known mutations, however, and the presence of LSCs is believed to be at least partially associated with resistance development and CML relapse. We previously proposed targeting quiescent LSCs as a therapeutic approach to CML, and a number of potential strategies for targeting insensitive LSCs have been presented over the last decade. The identification of specific markers distinguishing CML-LSCs from healthy HSCs, and the potential contributions of the bone marrow microenvironment to CML pathogenesis, have also been explored. Nonetheless, 25% of CML patients are still expected to switch TKIs at least once, and various TKI discontinuation studies have shown a wide range in the incidence of molecular relapse (from 30% to 60%). In this review, we revisit the current knowledge regarding the role(s) of LSCs in CML leukemogenesis and response to pharmacological treatment and explore how durable treatment-free remission may be achieved and maintained after discontinuing TKI treatment.

## 1. Introduction

Based on the American Cancer Society’s current estimates, 9110 new cases of chronic myeloid leukemia (CML) will be diagnosed in the United States every year, and 1220 patients will die of CML. The incidence rate of CML is 1–2 cases per 100,000 adults, and the average age at diagnosis is ~64 years (2021 estimates by American Cancer Society). CML is typically linked to the Philadelphia chromosome (Ph^+^), which results from the t(9;22)(q34;q11) balanced reciprocal translocation. The consequence of this translocation is the generation of the BCR-ABL1 fusion protein, endowed with constitutive kinase activity that is not only necessary, but also, importantly, sufficient for CML pathogenesis [1]. The prognosis of CML patients in the chronic phase (CP) has significantly improved over the last two decades, as small-molecule tyrosine kinase inhibitors (TKIs) have been integrated into CML therapy in the clinic. Historically, the median survival of CML patients was 3–5 years from time of diagnosis, and hematopoietic stem cell (HSC) transplantation was considered the only curative therapy; however, the life expectancy of CML patients is now approaching that of the general population [2,3]. As a result, CML is currently viewed as a chronic ailment rather than a potentially lethal disease. However, in most patients receiving TKIs, BCR-ABL1 transcripts are readily detectable. TKI monotherapies may not be able to completely eliminate Ph^+^ leukemic cells, and response in the accelerated (AP) or blastic phase (BP) of the disease is generally short-lived [4,5], leading to life-long treatment with TKIs. One of the current treatment goals for CML patients with stable deep molecular response (DMR; i.e., MR4.5, BCR-ABL1 ≤ 0.0032% on the international scale; MR4.0, BCR-ABL1 ≤ 0.01% on the international scale) is maintaining durable treatment-free remission (TFR) after discontinuing TKIs; this not only improves quality of life for CML patients, but also leads to reduce financial burdens for individuals and communities [3,6,7,8].

Multiple studies have conducted prospective clinical trials focused on imatinib withdrawal in CML patients, where the levels of *BCR-ABL1* in the peripheral blood were closely monitored by polymerase chain reaction (PCR). With discontinuation, 40–70% of patients were able to maintain remission and did not need to restart therapy. However, molecular relapse was observed in 30–60% of the patients even though the majority of the relapsing patients exhibited wild-type BCR-ABL [9,10,11,12]. Over 25% of CML patients acquire TKI intolerance or resistance and are required to switch TKIs at least once [13]. The best-studied mechanisms of TKI resistance are mutations in the kinase domain of BCR-ABL1 [14]. These mutations, however, cannot explain 20–40% of resistant cases of CML. Leukemic stem cells (LSCs) represent a rare subpopulation of leukemic cells, which possesses stem cell characteristics, including self-renewal and differentiation capacity, quiescence, and high drug efflux potential, many of which are shared with healthy HSCs [15,16]. LSCs have been typically defined by their functional properties to initiate and maintain leukemia in mouse and/or xenotransplantation models. Xenograft studies have shown that LSCs are not eliminated by chemotherapy and eventually prompt disease relapse [17,18]. Similarly, hematopoietic stem and progenitor cells harboring leukemia-associated mutations persist after anti-leukemic therapies in patients [19,20,21]. To achieve permanent cures and prevent relapse, eradication of LSCs is essential [22,23]. LSCs in the context of CML have never been clearly defined yet, either from an immunophenotypical or functional standpoint. The existence of BCR-ABL1-independent LSCs has been suggested as opposed to the BCR-ABL1-dependent bulk CML cells, and the insensitive LSCs to TKI treatment are also examined a driver of disease progression. In humans, CML LSCs are experimentally assessed by leukemic cells transferred to immune-deficient mice, the ability to exhibit leukemic engraftment that is maintained over serial transplantations, and differentiation into the committed progenies that are not able to initiate leukemia upon their transplantation. These LSCs could explain why TKIs fail to clear minimal residual disease even in responding patients, and why these patients experience recurrence upon TKI discontinuation while achieving deep molecular response [24]. In this review, in addition to revisiting the advances in our knowledge of the roles of CML LSCs in leukemogenesis, we will alternate between discussions of healthy control of HSCs and the deregulation that contributes to pathogenesis of the Ph^+^-related hematological malignancy of B-ALL.

## 2. Aberrant Cell Surface Antigens in CML LSCs

LSCs are known to express the hallmarks of stem cells, including (epi)genetic alterations and active efflux pumps, as well as deregulated cell signaling pathways that enhance self-renewal [25]. Cell-extrinsic microenvironmental factors provided by the bone marrow niche may also support their survival and/or contribute to lower sensitivity or resistance to TKIs. CML LSCs supposedly reside within the CD34^+^/CD38^−^/Lin^−^ fraction [26], and this immunophenotype is shared by healthy HSCs. The identification of unique cell surface antigens in CML has been a major challenge, but it is critically important to refine the enumeration of remaining CML LSCs within the CD34^+^/CD38^−^/Lin^−^ fraction, as well as explore their therapeutic targeting. Recent efforts have led to the identification of several markers, such as CD25 and CD44, most of which are significantly expressed in CML LSCs but are also detectable in healthy hematopoietic stem and progenitor cells (HSPCs) [27,28,29] (Table 1). In contrast, interleukin-1-receptor accessory protein (IL-1RAP) is selectively expressed in CML LSCs but not in HSCs from healthy individuals [30,31,32]. Increased expression of the IL-1R complex confers on CML LSCs selective growth and survival advantages over HSCs. Blocking IL-1 signaling through an IL-1R antagonist [32] and targeting IL-1RAP with antibodies resulted in a decreased number of leukemic cells at the stem cell level [31,33]. An RNA-seq study further identified a subpopulation of primitive CML cells expressing CD36 that is quiescent and insensitive to imatinib compared to the CD36-negative subpopulation. This finding holds therapeutic promise because CD36-targeting antibodies are capable of inducing cytotoxicity in CD36^+^ leukemic cells [34].

One aberrant surface marker appears to be a low-affinity receptor for IL-2, CD25 (or IL-2RA) [36]. CD34^+^/CD38^−^ LSCs express CD25 in >90% of patients with untreated CML. CD25 could be useful as a CML-LSC marker at diagnosis, and it has been shown that BCR/ABL1 TKIs downregulate STAT5- and CD25-expression in LSCs [38]. However, CD25 in LSCs is not CML specific, it is also seen in AML [23] and is occasionally found in healthy bone marrow HSCs. BCR-ABL1 monitoring is more sensitive than LSC phenotyping, and it is not known whether CD25 is an appropriate target for CML therapy due to its potential role as a tumor suppressor in leukemogenesis. Other surface markers expressed on CML LSCs include CD56 and CD93 [27,39]. CD93 is found selectively expressed on CD34^+^CD38^−^CD90^+^ cells when engraftment capacity is enriched in patient-derived xenograft (PDX) models. The CD93^+^ CML LSC population is not eradicated by TKI treatment. Patients in TFR who have experienced molecular recurrence have shown detectable CD93^+^ within CD34^+^CD38^−^CD90^+^ cells. CD93 is not detectable on CML LSCs, but rather on other cell types such as platelets and endothelial cells; it is therefore unlikely that CD93 is a potential therapeutic target. Additional studies will be needed to assess whether CD93 is a predictive biomarker for selecting CML patients at high risk of molecular recurrence after TKI discontinuation.

Another promising marker is CD26 (dipeptidyl peptidase-4, or DPP4). CD26 is an aberrantly expressed protease on the CML LSC surface, but it is not in CD34^+^ cells from patients with other myeloid neoplasms or healthy controls [35,40]. CD26^+^ CML LSCs derived from chronic-phase CML patients were capable of inducing BCR-ABL^+^ engraftment in NOD-SCID-IL-2Rγ^−/−^ (NSG) mice [35]. CD26 disrupts the C-X-C motif chemokine 12 (CXCL12)/CXCR4 axis by cleaving CXCL12 (also known as stromal cell-derived factor 1 (SDF-1)) to promote LSC mobilization into the blood circulation, and DPP-4 inhibitors reduced disease expansion by promoting LSC homing [35,41,42]. During successful treatment with imatinib, CD26+ CML LSCs decreased, and in the majority of these cases, CD26+ CML LSCs were undetectable in the peripheral blood. Notably, BCR/ABL1 or imatinib does not affect expression of CD26 in CML LSCs. However, in some cases, residual CD26+ CML LSCs were detected during the initial phase (3 months) of imatinib therapy, and many of these patients experienced disease relapse [35]. At diagnosis, substantial heterogeneity has been observed within the putative LSC population in the bone marrow of CML patients, and the concentration of CD26^+^ LSCs correlates with TKI resistance [43]. Prospective studies monitoring the kinetics of CD26^+^ LSCs during TKI treatment have detected circulating CD26^+^ LSCs in 66% of CML patients in the TFR phase, although this number was significantly decreased compared with initial diagnosis [35,44]. Clearly, it would be clinically beneficial to develop a method for accurately counting LSCs and establish an LSC threshold that would allow us to identify patient candidates for TKI drug discontinuation who could achieve a prolonged TFR without relapse.

## 3. Metabolic Regulation in CML LSCs

LSCs demand a tightly regulated metabolism [45,46]. Branched-chain aminotransferase 1 (BCAT1) transfers the α-amino group of branched-chain amino acids (BCAAs) to alpha-ketoglutarate (αKG), which is an essential co-factor of ten-eleven translocation 2 (TET2) [47]. As the overexpression of BCAT1 is found in LSCs in *TET2*/*Isocitrate dehydrogenase* (*IDH*) wild-type acute myeloid leukemia [48,49], BCAT1 has been proposed as a driver of LSC self-renewal through its reduction of TET2 activity. The mitochondrial control for leukemogenesis and LSC function has recently been highlighted, and the increasing findings have implied targets with clinical promise. Indeed, a single-cell RNA sequencing study showed that mitochondrial oxidative phosphorylation (OXPHOS)-related genes are upregulated in BCR-ABL^+^ LSCs compared to healthy HSCs within the same CML patient samples [50]. Our previous research used *p210^BCR^*^–^*^ABL^*-transduced murine CML models to show the critical roles of the tumor suppressor promyelocytic leukemia (PML) in LSCs [51,52]. PML was first identified as a component of PML-RARα fusion protein in acute promyelocytic leukemia [53,54,55,56], and in addition to its well-known functions in DNA damage response, apoptosis, and senescence, investigations over the last decade have identified PML as a regulator of metabolic pathways in stem cell compartments, including the hematopoietic system. We have shown that functional loss of the Pml axis in fatty acid metabolism reduces stem cell self-renewal and triggers excessive commitment of HSCs, resulting in HSC exhaustion [57]. An investigation of fatty acid oxidation (FAO) in breast cancer reported that PML enhances FAO through PPAR signaling by inducing the deacetylation of PPAR-γ coactivator 1α (PGC1α) by Sirtuin 1 (SIRT1) [58]. These findings imply that the metabolic cues regulated by PML could play a role in the disease-initiating capacity of LSCs, and notably, SIRT1 activates PGC1α and promotes mitochondrial electron transport chain (ETC) activity [59]. The SIRT1 is overexpressed in LSCs from CML patients, and inhibition of SIRT1 activates p53 signaling, leading to the enhancement of targeting of CML LSCs by TKI treatment [60]. Better understanding of PML and its associate pathways at the levels of both healthy and malignant stem cells will provide suitable pharmacologic targets and will clarify the metabolic requirement in leukemogenesis. In a recent example from our own work, we contributed to a collaborative exploration of mitochondrial involvement downstream of cytoplasmic Nucleophosmin 1 (NPM1c) in hematological disorders; this study demonstrated that NPM1c targets PML and promotes cell growth through blunting the biogenesis of PML nuclear bodies and weakening mitochondrial fitness [61]. Critically, actinomycin D (ActD) was found to induce mitochondrial stress, ROS production, restoration of PML nuclear bodies (NBs), senescence, and inflammation-mediated remodeling of the microenvironment, leading to therapeutic efficacy against NPM1c^+^ leukemia [61].

Autophagy functions as a means of cellular quality control to maintain homeostasis under normal and stress conditions and is induced as a source of metabolites during nutrient deprivation. The critical roles of autophagy in hematopoiesis and HSC aging have been extensively explored [62], and studies of mouse models and recent advances in genetic/metabolomic analyses have also highlighted important contributions of autophagy to leukemogenesis. In primary CML cells, TKI treatment activates an autophagic process, and pharmacological inhibition of autophagy potentiates TKI-induced cell death [63]. Autophagy-related 4B cysteine peptidase (ATG4B) is highly expressed in CD34^+^ cells from CML patients, and knockdown of *ATG4B* sensitizes these cells to TKI treatment [64]. Mitochondrial autophagy, or mitophagy, is a specific form of “macro”-autophagy for the selective clearance of damaged mitochondria. The PPARδ-FAO pathway activates mitophagy in HSCs, and our single-cell approaches have identified a link between HSC expansion and this quality control process of the mitochondria [65]. Researchers have observed similarities in the mechanisms through which healthy HSCs and LSCs are maintained, and these studies not only support the notion that this self-clearance system may be a key determinant of division balance, but also suggest potential strategies for inducing LSC exhaustion through key metabolic pathways.

Recognized modes of resistance in CML LSCs to TKIs include the following: (a) primary and secondary (cell selection under treatment pressure) mutations of BCR/ABL, affecting TKI binding to the BCR/ABL tyrosine kinase domain; (b) amplification of *BCR*/*ABL*; (c) enhanced activity of drug exporters; (d) quiescence; (e) mutations outside *BCR*/*ABL*, determining BCR/ABL-independent survival and proliferation (mutation-driven loss of “oncogene addiction”); and (f) BCR/ABL protein suppression in cells expressing the *BCR*/*ABL* gene [14,66]. There are thus both BCR/ABL-dependent and -independent mechanisms, and the latter case has been shown to be bound to extremely low oxygen tension, a condition reminiscent of the microenvironment of the bone marrow niche in vivo [67,68]. This hypoxic environment suppresses expression of BCR/ABL protein [69]. Incubation under extremely low oxygen tension allows for the selection of BCR/ABL protein-negative and TKI-insensitive CML LSCs, which can survive and cycle independently of BCR-ABL signaling, from the bulk of CML cells. This resistance is not linked to cell quiescence, and critically, pharmacological inhibition of hypoxia-inducible factor-1 (HIF-1) targets LSCs rather than HSCs [70,71]. In hypoxia, the Pasteur effect (an enhanced glucose consumption rate) leads to constant, extremely low concentrations of glucose, which enable BCR/ABL-independent LSC self-renewal and maintenance of TKI-resistant residual LSCs [72]. In contrast, single-cell RNA sequencing has shown that the genes associated with oxidative phosphorylation (OxPhos) are upregulated in resistant CML LSCs compared to non-malignant HSCs from the same patients’ bone marrow, and in general, OxPhos appears to play a key role in LSC maintenance and treatment resistance in myeloid leukemia [50]. Further exploration of metabolic control in stem cell physiology and malignancy will allow us to understand the involvement of OxPhos in the maintenance of CML LSCs, and more importantly, deploy it as a strategic tool to increase the efficacy of TKIs against treatment-naïve CML cells, and perhaps overcome TKI resistance.

## 4. Cellular Pathways Active in CML LSCs

Non-metabolic active signaling pathways can also contribute to the resistance of CML LSCs to TKIs, including the Sonic hedgehog, Wnt/βcatenin, PI3K/AKT, and JAK/STAT. The aberrant activation of Hedgehog (Hh) through the upregulation of Smoothened (Smo) is required to maintain CML LSCs during treatment with TKIs. Loss of Smo impairs the induction of CML by the BCR-ABL1 oncoprotein and causes depletion of CML LSCs. Exposure to a Smo inhibitor, cyclopamine, decreases LSC numbers and inhibits growth, supporting the critical roles of constitutively active SMO in disease progression [1,73]. The Wnt pathway is not only crucial for HSC homeostasis, but also plays a role in maintaining CML LSCs. LSCs exhibit aberrant activation of β-catenin in blastic phase (BP) CML patients, and β-catenin deletion leads to a profound reduction in the ability to develop BCR-ABL-induced CML in mice in vivo [74,75,76]. BCR-ABL physically interacts with β-catenin and stabilizes and stimulates β-catenin through activation of phosphoinositide 3 kinase (PI3K)/Akt signaling in BP-CML cells. This accelerated disease progression in a murine CML model [77,78,79]. β-catenin inhibition can therefore reverse TKI resistance in BP-CML cells, either with or without BCR–ABL kinase domain mutations, and has been shown to synergize with TKI both ex vivo and in vivo [80], supporting the potential utility of combined inhibition of β-catenin and BCR-ABL for therapy of CML, particularly TKI-resistant BP-CML.

BCR-ABL1 activates the PI3K/AKT pathway and deregulates activity of multiple transcription factors, such as nuclear factor kappa-light-chain-enhancer of activated B cells (NF-kB) and Forkhead box O (FoxO). FoxOs are important for healthy hematopoietic homeostasis [81], and a critical role has been reported for them in the maintenance of LSCs with the transforming growth factor-β (TGF-β)–FoxO pathway [82]. In a murine CML model, LSCs exhibited increased nuclear localization of Foxo3a and decreased Akt phosphorylation. TGF-β controls Akt activity and Foxo3a localization in CML LSCs, and Akt activity is suppressed despite BCR-ABL expression in vivo, leading to increased nuclear localization of Foxo3a [82]. The BCL6 protooncogene is a key effector downstream of FoxO in self-renewal signaling of CML LSCs through its repression of the ARF/p53 pathway. Peptide inhibition of BCL6 led to xenografted human CML cells failing to initiate leukemia in transplant recipients, and selectively eradicated CD34^+^CD38^−^ LSCs in patient-derived CML samples [83].

The inflammatory environment supporting LSC survival can be targeted by blocking IL-6, which is an activator of STAT3 through JAK1-mediated tyrosine phosphorylation [84,85,86]. In CML, inhibition of STAT3 was found to reduce LSC survival in TKI-resistant samples [29]. Other recent studies have shown that STAT3 activation via JAK1 kinase activity in primary murine und human CML cells persists despite TKI therapy [87], and upon in vivo TKI treatment, STAT3 expression is upregulated in human CD34^+^ CML cells. Proliferation of human CD34^+^ CML cells, however, was inhibited by combined inhibition of BCR-ABL and JAK1. These combinatory therapies induced apoptosis both in proliferating and quiescent human CML LSCs [87]. BCR-ABL1 thus activates multiple downstream pathways for the survival and/or self-renewal of LSCs, and some of these pathways are boosted by TKI treatment. Better understanding of the malignant activation of these signaling axes will lead to new therapeutic rationales for targeting them in addition to BCR-ABL for potentially curative CML therapies.

## 5. Targeting the Bone Marrow Microenvironment for CML LSCs

HSPCs interact with various types of bone marrow cells, including endothelial cells, neural cells, osteoclasts, mesenchymal stromal cells, and osteoblasts [88]. In addition to cell-intrinsic factors, hematopoietic homeostasis is regulated by an intricate set of cell-extrinsic cues from the bone marrow microenvironment, or niche. Endothelial cells are important niche constituents that form the vascular niche and produce various factors (e.g., CXCL12 or SDF-1, stem cell factor (SCF), Notch-ligand, and pleiotrophin) to regulate HSPC activity [89,90,91]. Dr. Paul Frenette and colleagues have shown that the combined action of endothelial selectins (P and E-) and CD106 (vascular cell adhesion molecule-1, or VCAM-1) promote the optimal recruitment and homing of HSPCs to the bone marrow [88,92], while integrins and CD44 support HSPCs by binding to endothelial cells and the extracellular matrix. Rolling of HSPCs is mediated by interaction between very late antigen 4 (VLA4) and P/E-selectins, while CXCL12 and its receptor CXCR4 act as a chemo-attractant and are critical for stable engraftment of HSPCs in the bone marrow niche [88].

**Figure 1:**
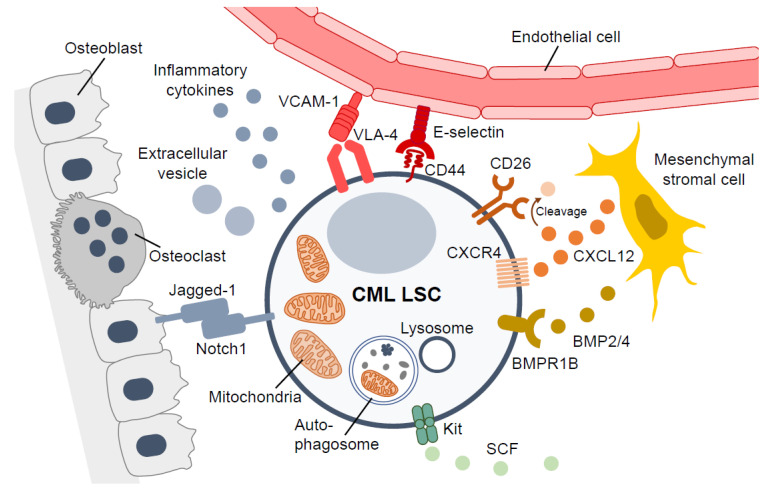
CML leukemic stem cells, and c of the bone marrow microenvironment for CML LSCs. CD26 disrupts the CXCL12/CXCR4 axis by cleaving CXCL12 to promote LSCs’ mobilization into the blood. LSCs bind to VCAM-1 and Selectin on their bone marrow niche. Secretion of LSCs demand a tightly regulated metabolism. BMP2/4 and other chemokines/cytokines through auto- or paracrine mechanisms also support quiescence, cellular growth, and drug resistance of CML LSCs. LSC, leukemia stem cell; VCAM-1, vascular cell adhesion molecule-1; VLA-4, very late antigen 4; CXCL12, C-X-C motif chemokine 12; SCF, stem cell factor; BMP, bone morphogenetic protein.

The occupation of the bone marrow niche by either healthy HSPCs or leukemic cells depends on differentiation status and disease progression [93]; it is therefore no surprise that LSC interaction with the bone marrow microenvironment has been described as a key component of leukemia pathogenesis (Figure 1). Indeed, the ectopic expression of BCR-ABL1 is known to impair the CXCL12-CXCR4 axis at this life stage of HSPCs; studies have found CXCL12 expression in the bone marrow stromal cells was reduced in both BCR-ABL transgenic mice and CML patients, while its level in the spleen was increased in CML mice compared to control mice [94]. The decrease of CXCL12 in bone marrow was partly mediated by the enhanced secretion of G-CSF by leukemic cells, while the increased expression of CD26 on CML LSCs interrupted CXCL12-CXCR4 interaction. These effects were shown to contribute to the impaired retention of immature hematopoietic cells in the bone marrow as well as the egress of LSCs toward their altered localization in the spleen, with the potential of uncontrolled extramedullary myeloproliferation in local LSC reservoirs [35,41,42].

A recent study of B-cell acute lymphoblastic leukemia (B-ALL) also identified contributions of the microenvironment to leukemia phenotypes. Ph^+^ is the most common cytogenetic abnormality in a subset of B-ALL, occurring in 2–5% of pediatric B-ALL and ~30% of adult B-ALL cases, and is associated with poor prognosis [95,96]. CML is rare in childhood but more prevalent among adults, due at least in part to increasing myeloid-biased hematopoiesis with age. Dr. Krause’s group explored the contributions of the aged microenvironment to CML pathogenesis [97]. C-X-C motif chemokine 13 (CXCL13) is a B cell chemo-attractant secreted by macrophages and is bound by its receptor CXCR5. Macrophages in young mice were found to produce higher levels of CXCL13 than those in old mice, and the CXCR5-CXCL13 axis promoted the proliferation of B-ALL cells in a young bone marrow microenvironment. Consistently, Cxcr5 deletion in B-ALL stem cells showed survival advantages in a B-ALL murine model, while high expression of CXCR5 in pediatric B-ALL predicted central nervous system relapse [97]. These data support the idea that the aging of bone marrow macrophages influences leukemia phenotype and highlight the CXCL13-CXCR5 axis as a potential target for B-ALL therapy.

The interaction of LSCs with the bone marrow microenvironment has been shown to contribute to the oncogenicity of TKI-resistant CML LSCs. For instance, pharmacological inhibition of the tyrosine kinase activity of BCR-ABL1 reduces CXCR4, which favors egress of CML LSCs into the blood circulation from the bone marrow niche [98]. Increased CXCR4 consistently supports proper homing of CML LSCs to the bone marrow niche, which induces quiescence and TKI resistance [98,99]. Upregulation of CD44, along with increased binding to E-selectin, is observed in BCR-ABL-expressing cells with the *T315I* mutation, and these cells, which adhere to stromal cells, are quiescent [37], suggesting that these alterations of extrinsic factors by CML LSCs promote exclusive LSC lodging and dormancy, allowing them to escape TKI targeting. Interestingly, both murine and human TKI-resistant leukemic cells with the *T315I* mutation exhibited different spatial locations and niche interactions in the bone marrow compared to *BCR-ABL1^+^* native cells. The increased expression of integrin β3 decelerated CML progression, while knockdown of *Integrin-linked kinase* (*Ilk*) led to an increase in fibronectin deposition by *T315I* mutant cells, with prolonged survival in xenogeneic and syngeneic murine transplantation models [100], offering an additional example of the therapeutic manipulation of the levels of extracellular matrix proteins (e.g., fibronectin) against TKI-resistant CML. Further, crosstalk between CML LSCs and their niches is also mediated through paracrine and autocrine mechanisms. For instance, CML LSCs establish a bone morphogenetic protein (BMP) autocrine loop to acquire TKI resistance; TKI-resistant LSCs have higher expression of BMPR1b and are associated with overproduction of BMPs by the microenvironment, such as mesenchymal stromal cells, through autocrine (and/or paracrine) mechanisms. This BMP/BMPR1b autocrine loop has no effect on BCR-ABL transcripts but is accompanied by increased TWIST1 expression, facilitating resistance of primitive CML cells to TKIs [101].

Leukemic cells are known to affect the bone marrow environment and contribute to deregulated cytokine levels [102]. In the pathogenesis of CML, pro-inflammatory cytokines, including IL-6, IL-1β, and TNF-α, are upregulated in the serum [103], and this “pro-inflammatory environment” may promote a selective advantage for LSCs. Indeed, IL-1 has been reported as a positive regulator of CML LSCs. An antibody-based therapy against IL1RAP efficiently targeted CML LSCs by blocking IL-1 signaling [31], while chronic exposure to IL-1 leads to exhaustion of healthy HSCs [104]. The pro-inflammatory environment thus includes features with therapeutic potential. To understand the important regulators of CML LSCs, the Fioretos group recently conducted a cytokine screen using primary CD34^+^CD38^-^ cells from CML-CP patients. This screen not only confirmed the known positive regulators of primitive CML expansion (e.g., IL-3, IL-1α/β, GM-CSF, IL-6, and IFN-γ), but also identified several new regulators, including myostatin propeptide (MSTNpp) [105]. Myostatin (growth and differentiation factor-8) is a member of the transforming growth factor-beta (TGF-β) superfamily. A better understanding of the pro-inflammatory environment, together with auto- and paracrine signaling, will be important in characterizing the development and progression of CML, and will help identify new therapeutic targets for CML LSCs.

The therapeutic efficacy of IFN-α against CML was first reported in the 1980s [106]. IFN-α therapy alone delayed disease progression and prolonged overall survival, and in the 1990s, IFN-α became a standard therapy for CML patients who were not suitable candidates for bone marrow transplantation [107]. TKI largely displaced IFN-α in clinical practice for CML, and in recent years IFN-α has mainly been used during pregnancy or for patients with TKI intolerance [108]. However, the unique activity and immunological effects of IFN-α against CML LSCs have recently been highlighted. Studies have shown that combined therapy with TKIs and IFN-α produces synergistic effects evidenced by deeper molecular responses and eradication of mutant CML cells [109], and IFN-α monotherapy allows for the discontinuation of imatinib because it maintains the molecular remission levels achieved by prior combined therapy with TKIs and IFN-α [110]. IFN-α targets cell cycle progression and activates apoptosis [111,112], thus sensitizing LSCs and helping to eliminate them. IFN-α also modulates immunological mechanisms, and particularly enhances anti-tumor immunity through activation of autoimmune cells, including natural killer (NK) cells, B and T cells, and antigen-presenting cells [113]. During IFN therapy, immune surveillance was boosted and cytolytic activity of NK cells against autologous CML blasts increased steadily [114,115].

IFN-α dose reductions improve combination therapy with TKIs and lead to better tolerance and higher molecular remission rates. For example, a Phase II study by the Nordic group evaluated the long-term effects of combining peg-IFNα with imatinib [116]. MMR rates in the combination arm were significantly higher at 12 months compared to imatinib monotherapy (82% vs. 54%, respectively). The large French SPIRIT trial observed faster and better molecular responses using imatinib + IFN-α treatment than treatment with imatinib alone (*n* = 159). Rates of molecular response were higher in the co-treatment group, and the majority of patients receiving peg-IFN for more than 12 months reached MMR (82%), while 49% achieved MR4 after 2 years, in contrast to patients treated only with imatinib (MMR, 43%; MR4, 21%, respectively) [117]. A German CML-Study IV randomized >1000 patients into three cohorts: (1) imatinib monotherapy 400 mg QD, (2) imatinib 400 mg QD plus IFN-α, and (3) imatinib 800 mg QD. Subsequent analysis demonstrated that imatinib plus IFN-α and standard imatinib (400 mg) therapy were comparable in outcome, while high-dose imatinib (800 mg) led to superior outcomes [118]. These discrepancies between the SPIRIT and Study IV results could be due to the longer half-life of the peg-IFN-α used in the SPIRIT trial [119]. Combined treatment with imatinib + IFN-α improves the depth and speed of remission, while toxicity concerns could result in more frequent termination of IFN-α treatment.

TKI resistance has driven the development of a new generation of TKIs [120]. Dr. Liu’s group reported that a CML patient harboring the BCR-ABL1 mutations (T315I and E255V) achieved successful DMR by dasatinib + IFN-α [121], and combination therapies were subsequently applied in clinical trials in CML patients. For example, in CML trial NCT01725204, dasatinib was combined with peg-IFN-α2b (Table 2). Response rates rose sharply after the addition of peg-IFN-α2b, with increasing MMR achieved over time (10% at 3 months, 84% at 12 months, and 89% at 18 months, respectively). The NCT01872442 trial assessed the safety and effectiveness of dasatinib with low dosage of peg-IFN-α2b as a first-line therapy for CML-CP patients, and a French NiloPeg study demonstrated the synergistic effects of peg-IFN-α2b and nilotinib [122,123]. A French PETALS study (NCT02201459) compared nilotinib with nilotinib + peg-IFN, and an interim analysis of CML-CP patients to determine cumulative MR4.5 rates one year after nilotinib initiation showed DMR rates favoring the combination treatment arm, which showed similar results in a PINNACLE study (Table 2). Interim analysis of the ongoing TIGER (CML V)-Study (NCT01657604) has shown that the rates of MR4.0 and MR4.5, which are associated with higher rates of TFR, can be improved by peg-IFN when added upfront to nilotinib [124].

## 6. Conclusions

The management of patients with CML has markedly improved since the introduction of TKIs, and the life spans of CML patients are now nearly indistinguishable from those of individuals without leukemia. Discontinuing TKIs for treatment-free remission has now become a clinical goal for CML, but the required eradication of all LSCs remains a challenge. We have just begun to understand how TKI-resistant clones evolve in CML patients, and how key pathways and mechanisms promote the survival of leukemic cells and/or LSCs. Despite the development of single-cell approaches to the study of healthy hematopoiesis, tracking individual LSCs continues to be challenging, even though the eradication of all single LSCs is essential for a cure of this disease. Recent advances in the identification of leukemia- and LSC-specific surface markers have allowed us to purify LSC fractions, and humanized xenograft models enable the analysis of human LSC clones in vivo. The development of accurate single-cell assay techniques for LSCs could lead to a better understanding of the molecular basis of LSC functions. Identifying the key metabolic and extrinsic cues that control LSC fate could yield effective targets for new strategies designed to achieve LSC eradication, which could produce significant benefits in the clinic.

## Figures and Tables

**Table 1:** Aberrant surface molecule expression in CML LSCs. LSC, leukemia stem cell; HSC, hematopoietic stem cell.

Surface Molecule	CML LSC	HSC	Reference
CD26 (DPPIV)	+	−	Herrmann et al. [35]
IL-1RAP	+	−	Landberg et al. [30]
CD36	+	−	Landberg et al. [34]
CD25 (IL-2RA)	++	+	Sadonvnik et al. [27], Kobayashi et al. [36]
CD44 (LHR)	++	+	Krause et al. [37]
CD34	+	+	
CD38	−	−	
Lineage Markers (Lin)	−	−	

**Table 2:** Candidate targets and clinical trials for CML patients.

Exemplar	Target	Clinical Trial Identifier	Outcome/Interim Analysis
Dasatinib + pegylated IFN	Immunity	NCT01725204	Increased MMR achieved over time (interim analysis)
Nilotinib + pegylated IFN	Immunity	NCT01397734 (TIGER)	Rates of molecular response is improved by peg-IFN (interim analysis)
		NCT02201459 (French PETALS)	DMR rates in favor of the combination treatment arm (interim analysis)
Gliptins with Nilotinib	DDPIV	2017-000899-28 (Phase I/II)	N/A
Dasatinib + SMO antagonist	Hedgehog	NCT01357655 (Phase II)	N/A
		NCT01702064 (Phase I)	N/A
Ruxolitinib + Nilotinib	JAK2	NCT02973711 (Phase I/II)	N/A
Arsenic trioxide + TKIs	PML	NCT01397734	N/A
Ruxolitinib + Nilotinib	JAK2	NCT00006091	N/A
